# A robust protocol for the generation of human midbrain organoids

**DOI:** 10.1016/j.xpro.2021.100524

**Published:** 2021-05-04

**Authors:** Alise Zagare, Matthieu Gobin, Anna S. Monzel, Jens C. Schwamborn

**Affiliations:** 1 Developmental and Cellular Biology, Luxembourg Centre for Systems Biomedecine (LCSB), University of Luxembourg, 6, Avenue du Swing, 4367 Belvaux, Luxembourg

**Keywords:** Cell culture, Neuroscience, Stem Cells, Cell Differentiation, Organoids

## Abstract

The lack of advanced *in vitro* models recapitulating the human brain complexity is still a major obstacle in brain development and neurological disease research. Here, we describe a robust protocol to derive human midbrain organoids from neuroepithelial stem cells. These complex 3D models are characterized by the presence of functional neurons, including dopaminergic neurons and glial cells, making them particularly attractive for the study of Parkinson disease.

For complete details on the use and execution of this protocol, please refer to Monzel et al. (2017).

## Highlights

• Reproducible generation of midbrain organoids from patterned neural precursor cells
• Midbrain organoids comprise dopaminergic neurons, astrocytes, and oligodendrocytes
• Cellular interaction and maturation are boosted by extracellular matrix embedding
• Suitable for *in vitro* midbrain development research and disease modeling

## Graphical abstract

**Figure:**
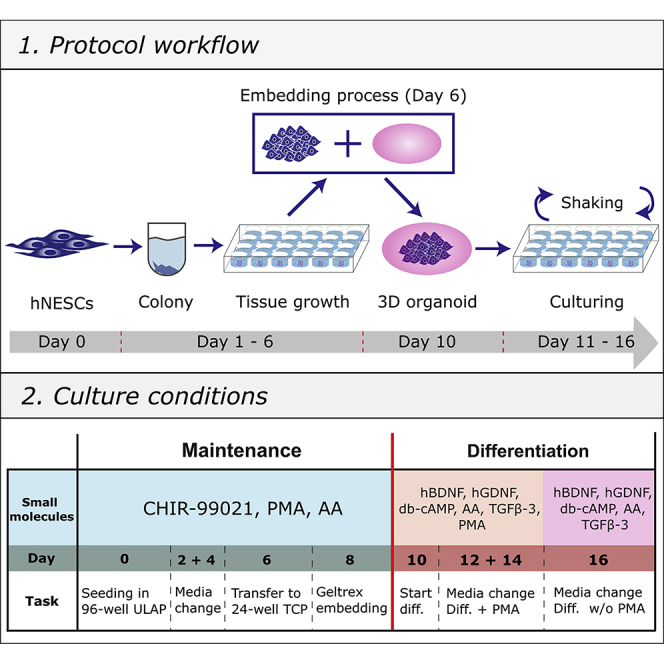

## Before you begin

***Note:*** All described protocol steps should be carried out under sterile conditions in a Class II biosafety cabinet and according to local regulatory requirements.

***Note:*** Cells are cultured throughout the whole procedure at 37°C with 5% CO_2_ in a humidified incubator.

### N2B27 base media preparation

**Timing: 20–30 min**

1. Prepare media using the recipe detailed in the Materials and Equipment section below.

***Note:*** N2B27 base medium is used as basis for the maintenance and differentiation media preparation. It can be stored at 4^0^C for up to one month. To prepare maintenance and differentiation media, small molecules should be added freshly on the day of media change in order to minimize small molecule degradation.

### Human neuroepithelial stem cell (NESC) maintenance

**Timing: 2 months (NESC derivation from iPSCs) or 2 weeks (if starting from a frozen NESC vial)**

**Figure 1:**
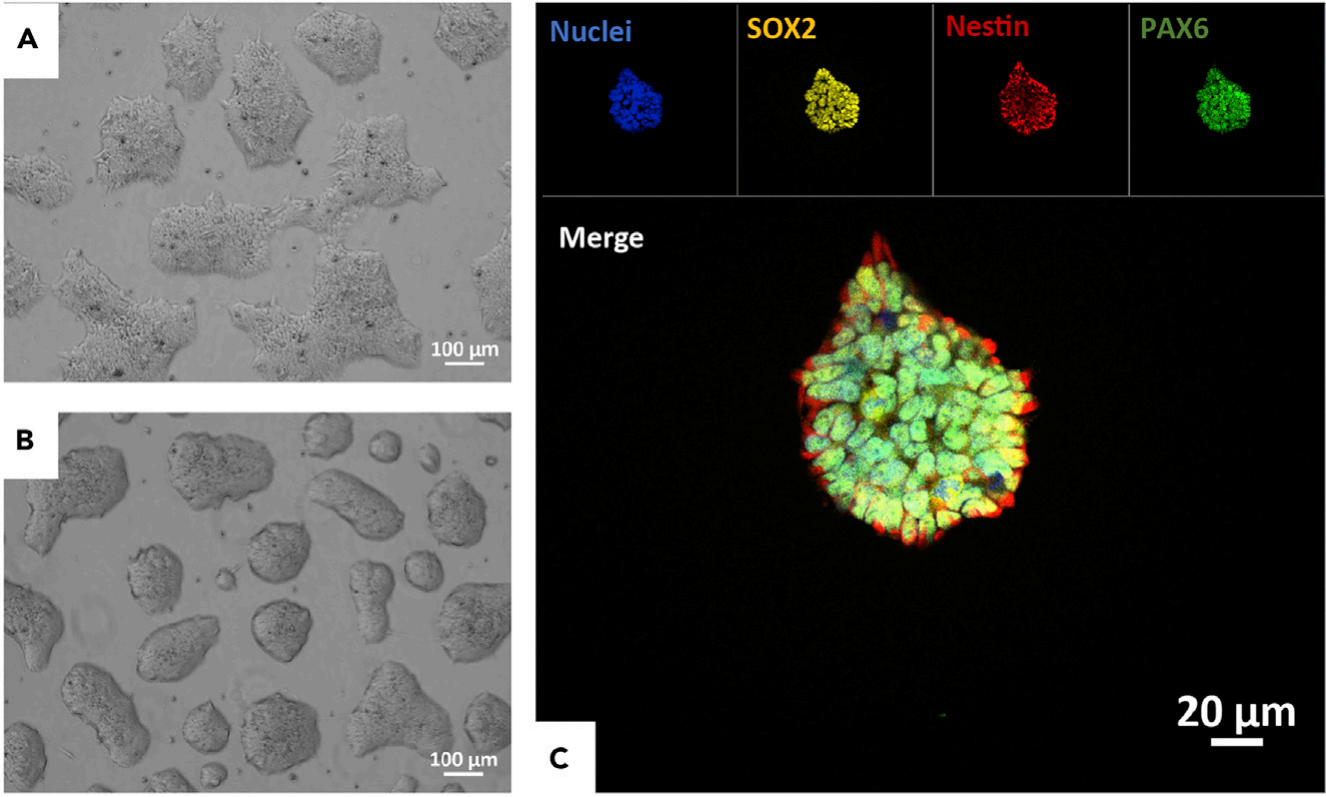
Representative images of NESC morphology and expression of neural progenitor markers (A and B) Bright-field image of two different NESC lines representing different colony morphology, either flat (A) or round (B). (C) Expression of the typical neural progenitor markers: SOX2 (yellow), Nestin (red), and PAX6 (green). Scale bar represents 100 μm (A and B) and 20 μm (C).

NESCs derived from human induced pluripotent stem cells (iPSCs), are the starting cell population for midbrain organoid generation (Figures 1A and 1B).

2. Derivation of NESCs
  a. NESC derivation is detailed in Reinhardt et al., (2013) in the Methods section at the paragraph *smNPC Derivation.* In brief, NESCs are derived from iPSCs via embryonic body (EB) formation. Expansion of neuroepithelium is achieved by exposing EBs to the small molecules CHIR99021 (CHIR, 3μM) and purmorphamine (PMA, 0.5μM) in order to stimulate the canonical WNT and SHH signaling pathways. Homogenous NESC culture shows stable expression of neural progenitor markers: Nestin, SOX2, and PAX6 (Figure 1C).

**CRITICAL:** Please be aware that to acquire homogeneous NESC culture after derivation from IPSCs takes about 2 months (seven to eight NESC passages). If the NESCs come from a collaborator, company or in-house stock as frozen vial, please refer to step 4.

3. NESCs are cultured in Matrigel- or Geltrex-coated 6-well plates.
  ***Note:*** Coated plates are prepared at least a day before cell seeding.
  a. Dilute Matrigel (1:100) or Geltrex (1:90) in cold (4°C) KnockOut DMEM. A total volume of 1.5 mL of diluted matrix is required to coat one well of a 6-well plate.
    **CRITICAL:** Thaw and keep Matrigel and Geltrex on ice during the dilution step because they polymerize at room temperature (18°C–22°C).
  b. Keep coated plate at room temperature for 24 h to stabilize the coating. Afterward, seal the plate with parafilm and put in the fridge at 4°C for storage.
    ***Note:*** Sealed plates can be stored at 4°C for up to one month.
  c. Before cell seeding, let the pre-coated plates equilibrate to room temperature.

4. Defreezing NESCs from a stock vial
  ***Note:*** If you derived NESCs as indicated in step 2, please skip this step.
  a. Thaw cells rapidly by immersing the vial in a water bath set at 37°C.
  b. Transfer the content of the vial to a 15 mL falcon.
  c. Add 9 mL of pre-warmed DMEM/F12 drop-by-drop. Use some of suspension to wash the cryovial once.
  d. Centrifuge for 3 min at 300 xg.
  e. Aspirate the supernatant and resuspend the cell pellet in 2 mL N2B27 maintenance medium.
  f. Transfer the whole suspension to a Matrigel- or Geltrex pre-coated wells (see the next step).

5. NESCs are routinely passaged at 80%–90% confluence using Accutase. Confluence is usually reached within 5–7 days.
  a. Remove the old media and add 1 ml of pre-warmed Accutase (37°C).
  b. Incubate for 4–6 min at 37°C in an incubator.
  c. Collect detached cells in 9 mL of DMEM/F12 to neutralize Accutase.
  d. Centrifuge for 3 min at 300 xg.
  e. Aspirate the supernatant and resuspend the cell pellet in 1 ml of freshly prepared N2B27 maintenance media.
  f. Count viable cells using Trypan Blue with a hemocytometer or an automated Cell counter.
  g. Prepare plate for cell seeding by removing Matrigel/Geltrex from the wells where cells will be seeded, and adding of 2 ml of N2B27 maintenance media in each well of 6-well plate.
  h. Seed 4–6 **×**10^5^ cells in prepared wells.
  i. Fully change media every 2^nd^ day.

***Note:*** After thawing, it usually takes 2–3 passages for the NESCs to fully recover from freezing and be ready for midbrain organoid generation. Before starting the organoid generation, NESC quality can be verified by confirming the expression of the neural progenitor markers Nestin, SOX2 and PAX6 (Protocol for staining is detailed in Reinhardt et al., (2013)).

### Equipment preparation

**Timing: 1 h**

6. Autoclave scissors and forceps for Day 5, which are needed to prepare the embedding materials (step 10) and at the embedding stage (step 11 and 12).
7. Sterilize a horizontal shaker before placing it in an incubator as detailed in step 15.

## Key resources table

**Table:** 

REAGENT or RESOURCE	SOURCE	IDENTIFIER
**Antibodies**		

Nestin (1:200 dilution)	BD Biosciences	611659
SOX2 (1:200 dilution)	R&D systems	AF2018
PAX6 (1:300 dilution)	BioLegend	901301

**Chemicals, peptides, and recombinant proteins**		

Matrigel® hESC-Qualified Matrix, ∗LDEV-free	Corning	354277
Geltrex hESC-Qualified	Invitrogen	A1413302
DMEM/F-12	Invitrogen	21331046
KnockOut DMEM	Thermo Fisher	10829018
Neurobasal Medium	Invitrogen	21103049
GlutaMAX Supplement	Thermo Fisher	35050061
N-2 Supplement	Thermo Fisher	17502001
B27 without Vitamin A	Invitrogen	12587-010
Penicillin/Streptomycin (P/S)	Invitrogen	15140122
CHIR-99021	Axon Medchem	CT 99021
Purmorphamine (PMA)	Enzo Life Sciences	ALX-420-045
Recombinant Human GDNF	PeproTech	450-10
Recombinant Human BDNF	PeproTech	450-02
Recombinant Human TGF-β3	PeproTech	100-36E
Db-cAMP	Sigma	D0627
Ascorbic acid	Sigma	A4544
Accutase	Sigma	A6964-500ML
Trypan blue	Invitrogen	T10282

**Experimental models: cell lines**		

Human iPSCs	Gibco	A13777

**Other**		

Ultra-low Attachment 96-Well Plate	Corning	7007
Non-treated 24-Well Plate	Celltreat	229524
Horizontal shaker	IKA	IKA KS 260 basic (0002980200)
Reagent reservoir	Gilson	F267660
Countess Cell Counting Chamber Slides	Invitrogen	C10283
Automated cell counter	Invitrogen	Countess™ II
Scissors and forceps	Hammacher	HWB 040-13 and DLM_HAMM_40271
6-Well plate	Thermo Fisher	140675

## Materials and equipment

### Media composition

**Table:** N2B27 base medium (store at 4^0^C up to one month)

Reagent	Final Concentration	Amount
DMEM HAM’s F12 medium	n/a	48 mL
Neurobasal medium	n/a	48 mL
GlutaMAX	2mM	1 mL
Pen/Strep	n/a	1 mL
N2	n/a	500 μL
B27 (without Vitamin A)	n/a	1 mL
**Total**		**100 mL**

**CRITICAL:** Please use B27 supplement without Vitamin A.

***Note:*** DMEM/F12 medium without any additives is also used in step 4 - *Before you begin* and step 8 - *Step-by-step method details.*

**Table:** N2B27 maintenance medium (prepare before use, do not store)

Reagent	Final Concentration	Amount
N2B27 base medium	n/a	10 mL
Ascorbic acid (20 mM)	150 μM	75 μL
CHIR (6 mM in DMSO)	3 μM	5 μL
PMA (10 mM in DMSO)	0.75 μM	0.75 μL
**Total**		**10 mL**

**Table:** N2B27 differentiation medium (prepare before use, do not store)

Reagent	Final Concentration	Amount
N2B27 base medium	n/a	10 mL
hBDNF (10 μg/mL)	10 ng/mL	10 μL
hGDNF (10 μg/mL)	10 ng/mL	10 μL
Ascorbic acid (20 mM)	200 μM	100 μL
TGFβ3 (10 ng/uL)	1 ng/mL	1 μL
db cAMP (100 mM in milliQ H_2_O)	500 μM	50 μL
PMA (10 mM)	1 μM	1 μL
**Total**		**10 mL**

**CRITICAL:** The small molecules are added to the N2B27 base medium right before the use. Please add PMA to the differentiation media just for the first six days of differentiation (Day 10–16). For long-term culture (> Day 16), use N2B27 differentiation media without PMA.

## Step-by-step method details

### Formation of single 3D colonies in a 96-well ultra-low attachment (ULA) plate (day 0–5)

**Timing: 6 days**

1. At 80%–90% of confluence, detach NESCs using Accutase as described in section *Before your begin (step 5).* Count viable cells via standard Trypan Blue protocol using a hemocytometer or an automated Cell counter.
2. Dilute 9 **×**10^5^ cells in 15 ml of N2B27 maintenance medium (calculated for 100 wells to have cell suspension in excess).

***Note:*** In each well, a total of 9 **×**10^3^ cells are seeded in 150 μL of N2B27 maintenance medium.

**CRITICAL:** Initial cell number for 3D colony generation has to be precise. We strongly advise to count multiple times and estimate the average number of viable cells.

**TROUBESHOOTING:** In case of inaccurate cell counting, please refer to Problem 1.

3. Distribute the 15 mL cell suspension in a sterile media reservoir.
4. Using a multichannel pipet, transfer 150 μL of cell suspension in each well of the 96-well ULA plate.

***Note:*** This is considered Day 0 of the protocol.

5. Change media every 2^nd^ day (Day 2 and 4) until Day 6.

**CRITICAL:** When changing media do not touch the colony by placing the tip to the bottom of the well.

We advise to remove 2/3 of the media by placing the tip on the side of the well and replacing with 150 μL of fresh N2B27 maintenance media.

**Figure 2:**
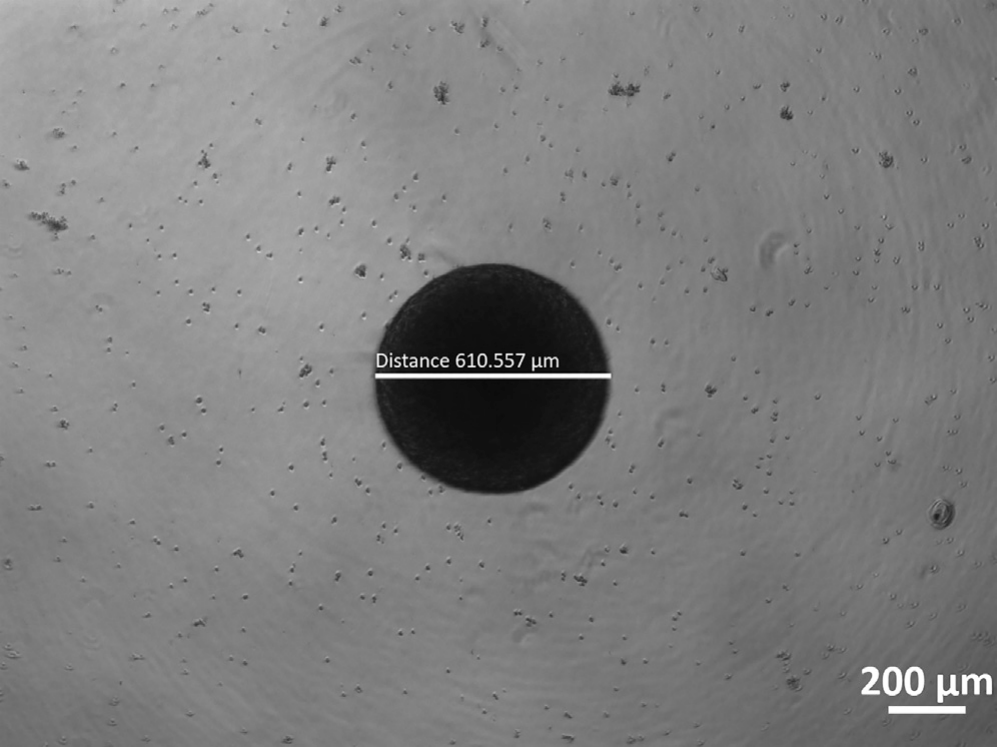
Representative picture of a single NESC 3D colony at day 6 showing minimal amount of debris and ready for embedding

***Note:*** Every day observe the colonies under the microscope. Single colonies should be formed within the first 2–3 days (depending on the cell line) with only a few cell debris left (Figure 2).

### Embedding material preparation (day 5)

**Timing: 1 h**

**Figure 3:**
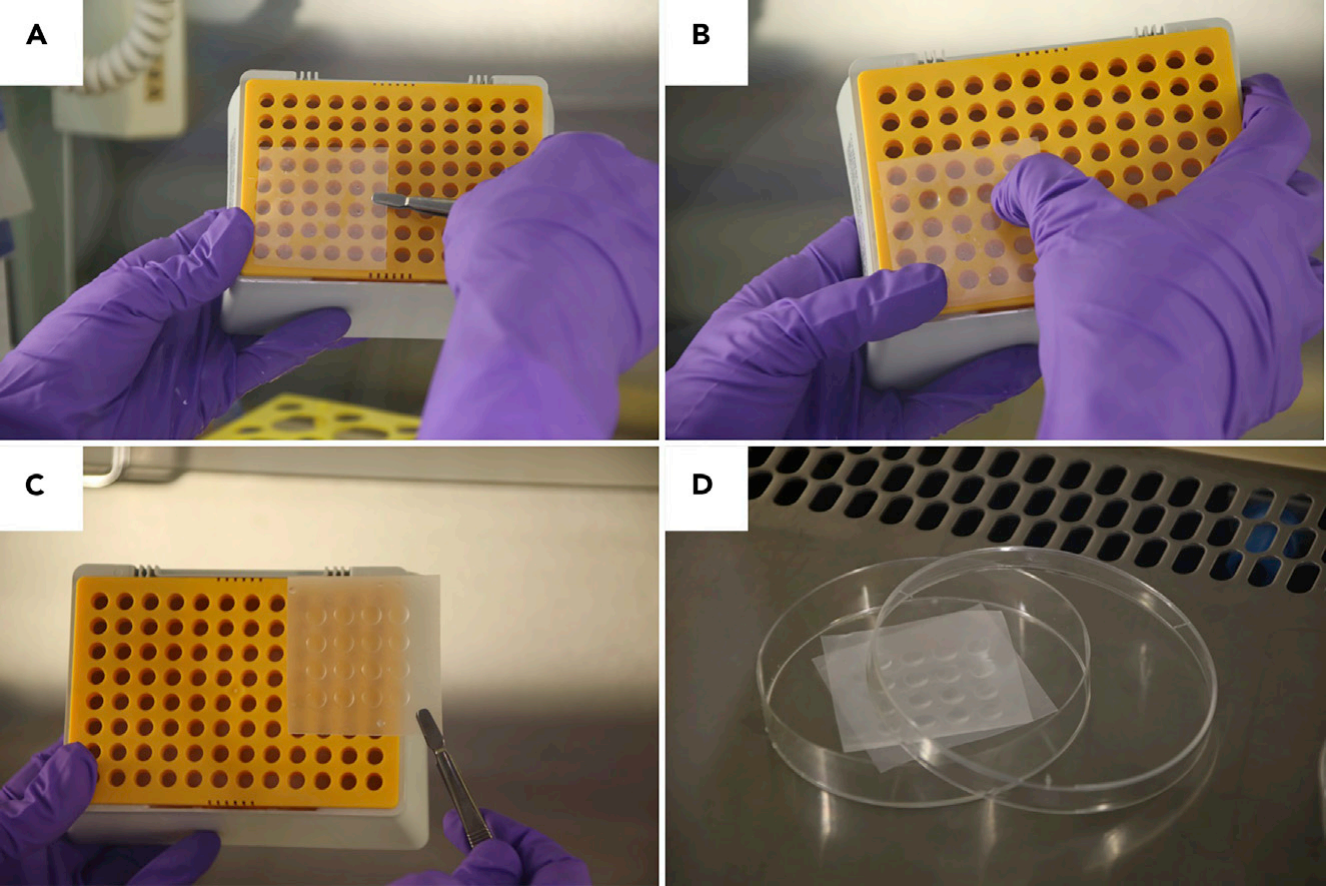
Preparation of dimpled parafilm The different steps highlighted are: (A) placement of the square-shaped parafilm on the tips box tray; (B) pressing the dimples by applying pressure on the parafilm above each hole; (C) completed 4**×**4 dimpled parafilm and (D) drying of the dimpled parafilm after immersion in ethanol and subsequent storing in a Petri dish.
  d. Create small dimples (4**×**4 dimples per Parafilm square) by pressing a gloved finger over the holes (Figures 3B and 3C).
    ***Note:*** You can either use an empty 200 or 1000 μL tip box/tray without the lid.
  e. Spray the dimpled parafilm square with 70% ethanol and using sterile forceps place it in a sterile 10 cm Petri dish in order for the ethanol to fully evaporate (Figure 3D).
  f. Once the parafilm is dry, close the Petri dish and seal it with Parafilm until embedding (step 12).
  g. Place the Geltrex stock vial on ice at 4°C in the fridge and let it thaw overnight (8–14 h).

***Note:*** Embedding in extracellular matrix supports neurite outgrowth and development of a more complex neuronal morphology.

6. Material preparation a day before the embedding
  a. Cut 6 Parafilm squares approx. 5**×**5 cm big (enough for one 96-well plate), spray them with 70% ethanol and place under the biosafety cabinet.
  b. Remove the parafilm from the paper and allow the ethanol to evaporate.
  c. Using sterile forceps, place one square of Parafilm over an empty tip tray (Figure 3A)

***Alternatives:*** Matrigel can also be used for embedding with the same protocol described here. Importantly, we have not observed any difference in organoid development between both alternatives. However, for result reproducibility we strongly advise to only use one brand of matrix (Matrigel or Geltrex) and adhere to the same throughout the whole experiment.

### 3D colony embedding (day 6)

**Timing: 3–4 h (for a single 96-well ULA plate)**

***Note:*** Throughout the following steps, sterile forceps and scissors are used (see Before you begin)

7. At day 6 of organoid culture, transfer the 3D colonies to an untreated 24-well tissue culture plate (TCP).
  a. Distribute 500 μL of pre-warmed (37°C) N2B27 maintenance medium to each well of the TCP.
  b. Cut a 1 ml pipet tip using sterile scissors and transfer each single 3D colony taken from the 96-well plate to a single well of the 24-well TCP (i.e., transfer one single 3D colony from one well to another).

***Note:*** Cutting the tip allows easier and gentler aspiration of the 3D colony.

**CRITICAL:** This step helps to clean the 3D colony from cellular debris and reduces the possibility for side colonies to be present in the upcoming embedding process.

**TROUBLESHOOTING:** If there is a presence of a side colony in one of the wells, please refer to Problem 2.

**Figure 4:**
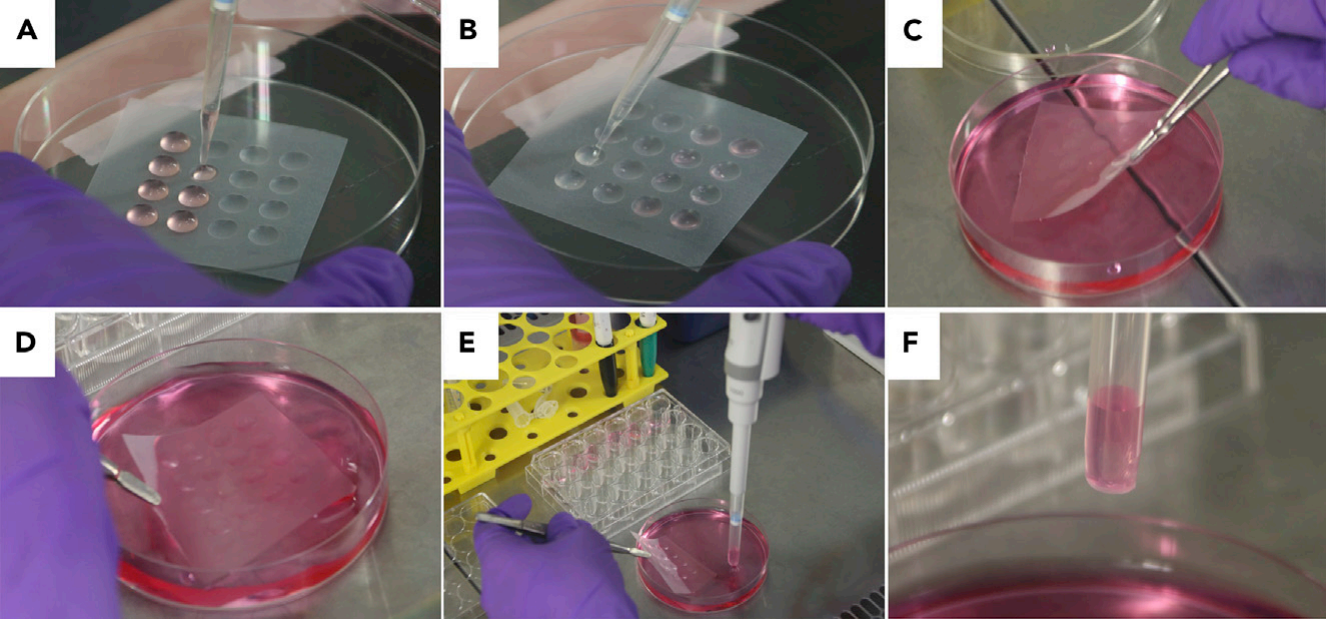
Step by step instruction of the colony embedding process (A–D) (A) Transfer of a single colony from the TCP to a dimple; (B) addition of the Geltrex to each colony after aspiration of the residual culturing medium; (C) placement of the polymerized Geltrex/colony droplets in medium and (D) shaking process in order to detach the embedded colonies from the parafilm. (E and F) Transfer of the embedded colonies in individual wells (24-well plate) using a 1 mL pipet with a cut tip.
  c. Remove the medium around the 3D colony using a 10 μL pipet tip.
    **CRITICAL:** Remove as much medium as possible to avoid Geltrex dilution. Be careful, not to leave the colonies to dry out on the dimpled parafilm.
  d. Add 35 μL of Geltrex around the 3D colony and try to keep the colony in the center of the Geltrex droplet (Figure 4B).
    ***Note:*** Place Geltrex on top of the 3D colony. If colony is not in the center, gently aspirate some Geltrex from one side of the droplet and add the aspirated amount to the other side of the droplet in order to push the 3D colony more to the center.
    **CRITICAL:** Avoid touching the 3D colony with the pipet tip as this would destroy it or lead to the formation of side colonies.
    **TROUBLESHOOTING:** If a colony drops out of the Geltrex droplet or is not centered, please refer to Problem 3.
  e. Incubate at 37°C for 25 min to allow the Geltrex to polymerize.
  f. Collect the Geltrex droplets with the embedded 3D colonies.
    i. Flip the Parafilm with the embedded colonies in order to immerse them into a new Petri dish filled with pre-warmed (37°C) DMEM/F12 (Figure 4C).
    ii. Incubate at 37°C for 25 min.
    iii. Remove the Geltrex droplets from the Parafilm by shaking slightly until the droplets fall off by themselves (Figure 4D).
  g. Using a cut 1 ml pipet tip, transfer a single embedded colony per well in a 24-well TCP (Figures 4E and 4F).
    ***Note:*** Ideally, you can use the same TCP plate as in step 7.
  h. Replace DMEM/F12 with 500 μL per well of N2B27 maintenance medium (see ‘Materials and Equipment’).
  i. Keep organoids in an incubator under static conditions until the differentiation (step 11).

8. Embed the NESC colonies in a Geltrex droplet.
  **CRITICAL:** Always use sterile forceps to handle the Parafilm squares and sterile scissors to cut the pipet tips.
  a. Place one dimpled Parafilm square in a fresh Petri dish.
  b. Place a single 3D colony from the TCP into each dimple using a cut 1 mL pipet tip (Figure 4A).

### Organoid differentiation (day 10)

**Timing: 6 days**

9. At the 2^nd^ day after embedding (Day 10 of organoid culture) replace N2B27 maintenance media with N2B27 differentiation media + PMA (see ‘Materials and Equipment’, 500 μL per well)

***Note:*** During media change, always try to remove as much media as possible before adding the fresh one. Since the matrix can be fragile and organoids are still small a few days just after embedding, media change can be done with a 1ml pipet by gently aspirating media and then adding a fresh one. After initiation of differentiation (from Day 10 on), neurite outgrowth stabilizes the matrix and organoids grow bigger, then a pump can be used to aspirate the media.

10. At day 12 of organoid culture, proceed to a full media change using fresh N2B27 differentiation media + PMA (see ‘Materials and Equipment’, 500 μL per well)
11. At day 14 of organoid culture, fully change media to fresh N2B27 differentiation media + PMA (see ‘Materials and Equipment’, 500 μL per well) and place the organoids on a horizontal shaker set at 80 rpm inside the incubator.

**CRITICAL:** Shaking is an essential step for optimal nutrient supply/exchange as well as organoid growth.

***Note:*** Sterilize the shaker before putting it in the incubator. We strongly advise to dedicate an incubator with shakers inside only to be used for this step.

**TROUBLESHOOTING:** If there is a side colony in the same Geltrex droplet, please refer to Problem 2. If the organoid is close to the edge of the Geltrex droplet, please refer to Problem 4. Lastly, if the organoid falls out of the Geltrex droplet, please refer to Problem 5.

12. At day 16 of organoid culture, proceed to a full media change using fresh N2B27 differentiation media without PMA (500 μL per well)

**CRITICAL:** At this stage, be aware to leave out the PMA for subsequent media changes. This stabilizes midbrain identity after initiation of differentiation (for further information, please refer to Reinhardt et al., 2013).

13. For long term culture, full media change using 500 μL of N2B27 differentiation without PMA is done every 3–4 days.

**CRITICAL:** Along with organoid development, nutrient consumption and cell metabolic activity increases. Visually we can see that when media becomes yellow before the regular media change (every 3–4 days). This is an important indicator to increase the volume of fresh media for the next media changes. Our observation shows that until day 30, organoids can be cultured in 500 μL per well. After this time, media volume should be increased to 600 μL per well in order to ensure optimal culture conditions.

## Expected outcomes

The primary outcome of the protocol is the generation of midbrain organoids, which represent complex 3D *in vitro* models that are suited for in-depth disease modeling. In particular, our model has attractive applications for *in vitro* modeling of diseases affecting the human midbrain area such as Parkinson disease (Nickels et al., 2020; Smits et al., 2019; Smits et al., 2020).

**Figure 5:**
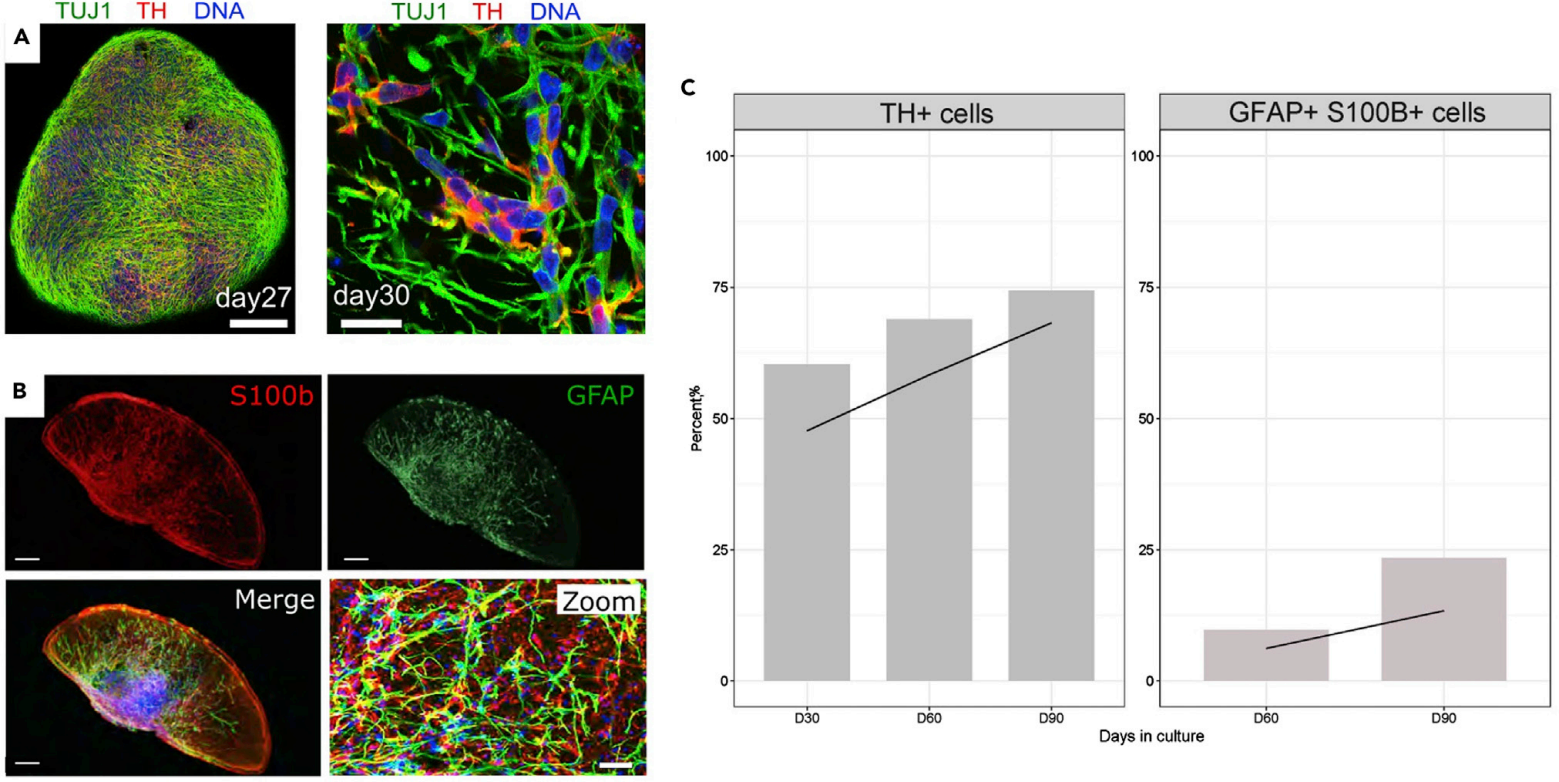
Representative immunofluorescent (IF) images of the neuronal and glial cell populations in midbrain organoids and their expansion during organoid development (A) Whole-mount IF staining for neuronal marker TUJ1 (green) and midbrain dopaminergic marker TH (red) at day 27 of organoid culture (left panel) and a representative image of TUJ1 and TH staining in a 50 μm section of 30 days old organoid (right panel). Nuclei are highlighted in blue. Scale bars, 200 μm (left) and 20 μm (right) (adapted with permission from Monzel et al., 2017). (B) The presence of astrocytes in midbrain organoids at day 60 of organoid culture is represented by the S100β (red) and GFAP (green) positive staining. Nuclei are depicted in blue. Scale bars represent 200 μm (main images) and 10 μm (zoom). (C) Representative histogram of the change in percentage of dopaminergic neurons (TH-positive, left) and glial cells (GFAP- and S100 b-positive, right) over the time (day 30, 60 and 90).

Organoids derived using the presented protocol harbor multiple brain cell types thus closely recapitulating the human brain environment, specifically the midbrain. Importantly, at day 30 of differentiation, organoids show a considerable TH-positive dopaminergic neuronal population (Figure 5A), which represents about 60% of the total live cell number (Figure 5C, left panel). Additionally, we observe that the amount of dopaminergic neurons increases steadily over time along with the organoid growth. Around day 60 of organoid culture, we can detect the presence of GFAP- and S100b-double positive astrocytes (Figure 5B). This glia cell population shows a strong increase over time reaching about 20% of live cells at day 90 of organoid culture. Furthermore, we have demonstrated the presence of mature oligodendrocytes forming myelin sheets in 61 days old midbrain organoids (Monzel et al., 2017).

The derived midbrain organoids are also suitable for functional analyses, as we were able to show the production of dopamine and the spontaneous activity of neurons recorded by multi-electrode array, confirming neuronal functionality (Monzel et al., 2017). These observations are essential indicators that the generated organoids recapitulate the human midbrain and also provide means for *in vitro* functional analysis of brain features. Lastly, the organoids handle well long-term culturing (up to one year), which is particularly suitable for modeling aging or prolonged drug testing.

In summary, our presented protocol generates a highly reproducible organoids in terms of size and cellular/molecular features which is paramount to provide a human midbrain-specific *in vitro* model.

## Limitations

Despite recent advances, the different organoid generation systems still need to be optimized in order to accommodate the complete cellular profile of the human brain. The integration of immune cells, mainly the resident macrophages of the brain called microglia, is still lacking in brain organoids. The major hurdle is that these cells are generated from a different cellular lineage and cannot be derived through the same procedure as neuronal cells. Thus, in recent years, growing efforts are undertaken to add this additional complexity level to brain organoids, which is an important step in understanding the role of these cells in various neurodegenerative diseases such as PD (Song et al., 2019).

**Figure 6:**
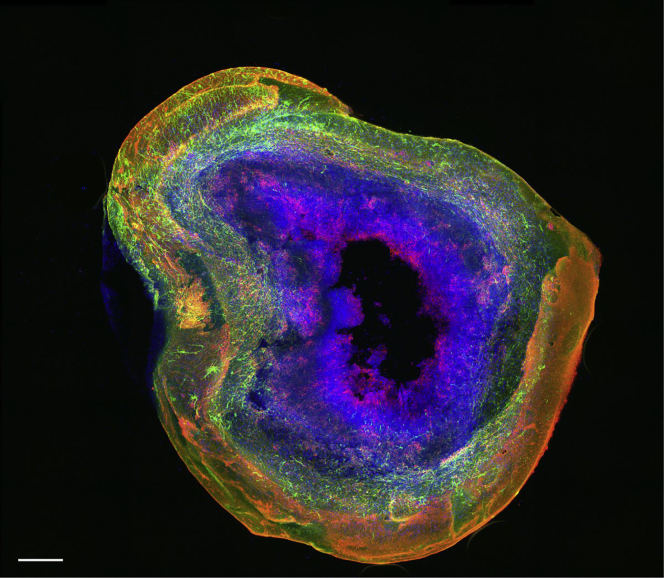
Representative picture of an organoid with substantial dead/necrotic core The section stained with the glia markers GFAP (green), S100B (red) and nuclei (blue) shows substantial dead/necrotic core in the center (black). Scale bar represents 200 μm.

Additionally, as the midbrain organoids are able to grow in size, they accumulate a so-called necrotic core in the center of the organoid. The core is comprised of dying or dead cells and builds up due to the increasing deficiency of oxygen and nutrient diffusion at the center of the organoid (Figure 6). In order to limit this phenomenon common to 3D cultures, the integration of functional vasculature around and/or in the organoid is a major undertaking in the community (Cakir et al., 2019; Shi et al., 2020).

## Troubleshooting

### Problem 1

If the cell count or distribution into the plates is not precise, the organoids will grow to be non-homogenous in size (extreme case either very small or very big; step 2).

### Potential solution

Although this will not affect the midbrain organoid phenotype but depending on the downstream experiments, discrepancies in size can influence results, (e.g., metabolic activity readings) and have to either i) be accounted for by the researcher, ii) used in bulk analyses (e.g., WB), which are less affected by size differences or iii) start over the protocol.

### Problem 2

**Figure 7:**
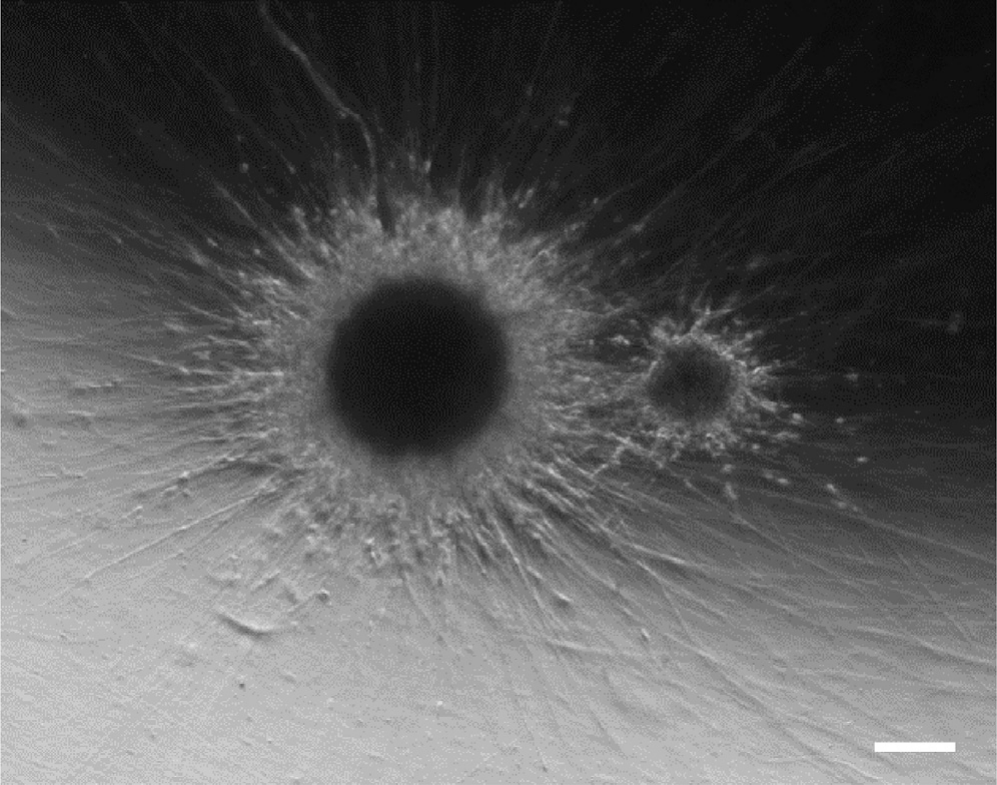
Example of a side colony present in the same Geltrex droplet Scale bar represents 200 μm.

Multiple or side colonies formed in the same well at any stage of organoid culture or in the same Geltrex droplet after embedding (Figure 7; step 7).

### Potential solution

Do not consider this well for embedding (in the case of multiple NESC colonies in the same well) or discard the Geltrex droplet with multiple embedded colonies because of reproducibility issues.

### Problem 3

NESC 3D colony falls out or is not centered in the Geltrex droplet during the embedding stage (step 12).

### Potential solution

Re-embed the colony using the same embedding steps by gently aspirating some Geltrex from one side of the droplet and add the aspirated amount to the other side of the droplet in order to push the 3D colony more to the center.

### Problem 4

**Figure 8:**
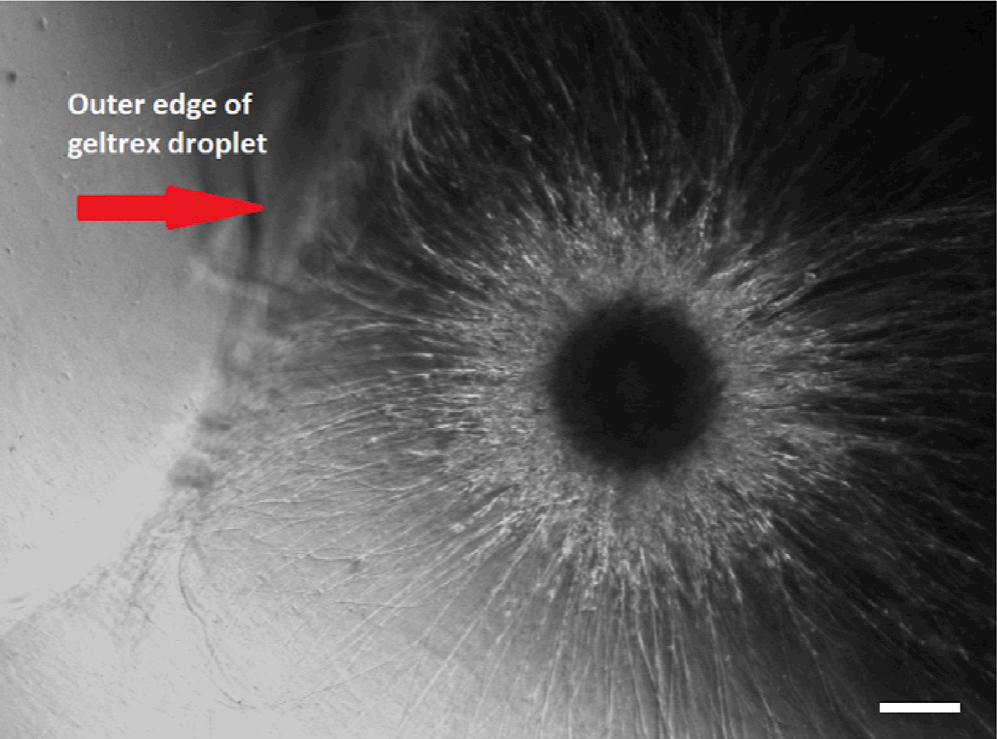
Example of an organoid not centered and very close to the edge of the Geltrex droplet Scale bar represents 200 μm.

Not properly (i.e., not in the center of the droplet) embedded colony in the Geltrex droplet (Figure 8; step 8).

### Potential solution

If organoid is on the edge of the Geltrex droplet then discard it, because it impairs the 3D structure and neurite outgrowth of the organoid.

If the organoid is not in the center but still surrounded by Geltrex then it can potentially be kept in culture and used for subsequent bulk experiments such as protein or nucleic acid analysis.

### Problem 5

Colony falls out of the Geltrex droplet during culturing (step 9).

### Potential solution

Discard the colony.

## Resource availability

### Lead contact

Further information and requests for resources and reagents should be directed to and will be fulfilled by the lead contact, Jens C. Schwamborn (jens.schwamborn@uni.lu).

### Materials availability

This study did not generate any new unique reagents or cell lines.

### Data and code availability

There is no dataset/code associated with the protocol.
